# Stage I Clear Cell and Serous Uterine Carcinoma: What Is the Right Adjuvant Therapy?

**DOI:** 10.3390/curroncol30010090

**Published:** 2023-01-14

**Authors:** Manon Lefebvre, Mathilde Duchatelet, Houssein El Hajj, Antoine De Courrèges, Jennifer Wallet, Charlotte Bellier, Florence Le Tinier, Marie Cécile Le Deley, Carlos Martinez Gomez, Eric Leblanc, Fabrice Narducci, Delphine Hudry

**Affiliations:** 1 Department of Gynecologic Oncology, Centre Oscar Lambret, 59000 Lille, France; m-duchatelet@o-lambret.fr (M.D.); houssein.elhajj@curie.fr (H.E.H.); c-martinezgomez@o-lambret.fr (C.M.G.); e-leblanc@o-lambret.fr (E.L.); f-narducci@o-lambret.fr (F.N.); d-hudry@o-lambret.fr (D.H.); 2 Methodology and Biostatistics Department, Oscar Lambret Comprehensive Cancer Center, 59020 Lille, France; a-decourreges@o-lambret.fr (A.D.C.); j-wallet@o-lambret.fr (J.W.); m-ledeley@o-lambret.fr (M.C.L.D.); 3 Department of Medical Oncology, Centre Oscar Lambret, 59000 Lille, France; c-bellier@o-lambret.fr; 4 Academic Department of Radiation Oncology, Oscar Lambret Comprehensive Cancer Center, 59020 Lille, France; f-letinier@o-lambret.fr; 5 Univ. Lille, Inserm, CHU Lille, U1192—Protéomique Réponse Inflammatoire Spectrométrie de Masse—PRISM, 59000 Lille, France

**Keywords:** clear cell uterine carcinoma, serous uterine carcinoma, stage I, survival, toxicities

## Abstract

This single-center study aimed to retrospectively evaluate the survival outcomes of patients with FIGO stage I clear cell and serous uterine carcinoma according to the type of adjuvant treatment received. The data were collected between 2003 and 2020 and only patients with stage I clear cell or serous uterine carcinoma treated with primary surgery were included. These were classified into three groups: No treatment or brachytherapy only (G1), radiotherapy +/− brachytherapy (G2), chemotherapy +/− radiotherapy +/− brachytherapy (G3). In total, we included 52 patients: 18 patients in G1, 16 in G2, and 18 in G3. Patients in the G3 group presented with poorer prognostic factors: 83.3% had serous histology, 27.8% LVSI, and 27.8% were FIGO stage IB. Patients treated with adjuvant radiotherapy showed an improved 5-year overall survival (OS) (*p* = 0.02) and a trend towards an enhanced 5-year progression-free survival (PFS) (*p* = 0.056). In contrast, OS (*p* = 0.97) and PFS (*p* = 0.84) in the chemotherapy group with poorer prognostic factors, were similar with increased toxicity (83.3%). Radiotherapy is associated with improved 5-year OS and tends to improve 5-year PFS in women with stage I clear cell and serous uterine carcinoma. Additional chemotherapy should be cautiously considered in serous carcinoma cases presenting poor histological prognostic factors.

## 1. Introduction

Papillary serous carcinoma (UPSC) and clear cell carcinoma (UCCC) are rare, high-risk, histological subtypes, accounting for 10 to 15% of all endometrial cancers. Due to their aggressive histologies, they are associated with poor outcomes and contribute significantly to endometrial cancer-related mortality, accounting for 40 to 50% of the deaths [1,2,3]. The primary treatment for early-stage uterine carcinoma remains surgery for both staging and therapeutic ends. This surgery consists of total hysterectomy with bilateral salpingo-oophorectomy and lymph node stadification [4,5,6]. Despite the aggressiveness and the increased risk of recurrence for these subtypes, optimal adjuvant therapy remains controversial, and data on the subject are scarce due to the low number and the heterogenous retrospective cohorts [5,7,8,9]. Consequently, some of these studies reached contradictory conclusions [10,11,12,13,14,15] and the international guidelines such as the National Comprehensive Cancer Network (NCCN) present substantial variability in the treatment options [16].

In the last ESGO (European Society of Gynecologic Oncology) recommendations for managing patients with endometrial carcinoma, concurrent chemo-radiation or sequential adjuvant chemotherapy and radiotherapy are recommended [17]. However, they highlight that the benefit of adding chemotherapy for patients with stage I–II UCCC remains unclear. Due to the rarity of patients presenting UCCC (1 to 6% of all endometrial cancers), it has been challenging to study this histologic subtype and determine a specific adjuvant therapeutic strategy [6,7,8]. 

Thus, the main objective of this study was to determine the impact of adjuvant therapy on survival outcomes of patients with stage I UPSC and UCCC. The secondary objective was to evaluate the toxicity related to each treatment type.

## 2. Materials and Methods

This is a single-center retrospective analysis performed at a high-volume tertiary cancer center.

The study was conducted according to the Declaration of Helsinki and approved by the Institutional Review Board (or Ethics Committee) of the Oscar Lambret Cancer Center. Ethical review and approval were waived for this study because it complies with the reference methodology MR004 adopted by the French Data Protection Authority (CNIL). None of the patients had objected to the use of their clinical data for research purposes. Of the included patients, three were excluded for missing consent. An institutional database was used to identify the cases.

All patients diagnosed with FIGO stage I UPSC or UCCC and treated with upfront hysterectomy and salpingo-oophorectomy between 2003 and 2020 were included. Patients with endometrioid carcinoma or mixed carcinoma were excluded.

Clinical and pathological data were collected based on a predefined dictionary. The following data were included: patients’ demographics, post-operative FIGO stages, surgery type, complications, histopathology results, adjuvant therapy and the associated complications, interval times between diagnosis and surgery and between surgery and adjuvant treatment, and survival outcomes.

Disease stage was determined using the 2009 FIGO classification for endometrial carcinoma [18]. According to Gynecological Oncology Group (GOG) pathology committee recommendations, only patients with tumors presenting a 50% or more clear cell component were included in the UCCC group.

We used the Clavien Dindo classification [19] to stratify perioperative morbidity. Complications and toxicities related to adjuvant therapy were collected and stratified according to the CTCAE classification [20].

Survival outcomes included progression-free survival (PFS), overall survival (OS), and survival after recurrence.

The patients were then classified into three groups based on the type of adjuvant therapy they received: No treatment or brachytherapy only (Group G1), radiotherapy +/− brachytherapy (Group G2), and chemotherapy +/− radiotherapy +/− brachytherapy (Group G3).

Median follow-up time was estimated by the reverse Kaplan–Meier method. Quantitative variables were described by means and standard deviations or medians and interquartile range. Frequencies and percentages described qualitative variables. Missing data were specified. The chi-square test and the student’s test were used to compare qualitative and quantitative variables, respectively. In cases of non-normality of the data, non-parametric Fisher’s exact test (qualitative variables) and Kruskal–Wallis test (quantitative variables) were used. OS and PFS were estimated using the Kaplan–Meier method and the different groups were compared using Cox proportional hazards models adjusted on a propensity score calculated beforehand. The significance level was set at 0.05. Cox proportional hazard regression models were used to identify significant prognostic factors on univariable analysis. Multivariate analysis was not performed because only one prognostic factor (age at diagnosis) reached the significance level of 0.2 on univariate analysis. Statistical analysis was performed using Stata v15.1 software.

## 3. Results

**Figure 1:**
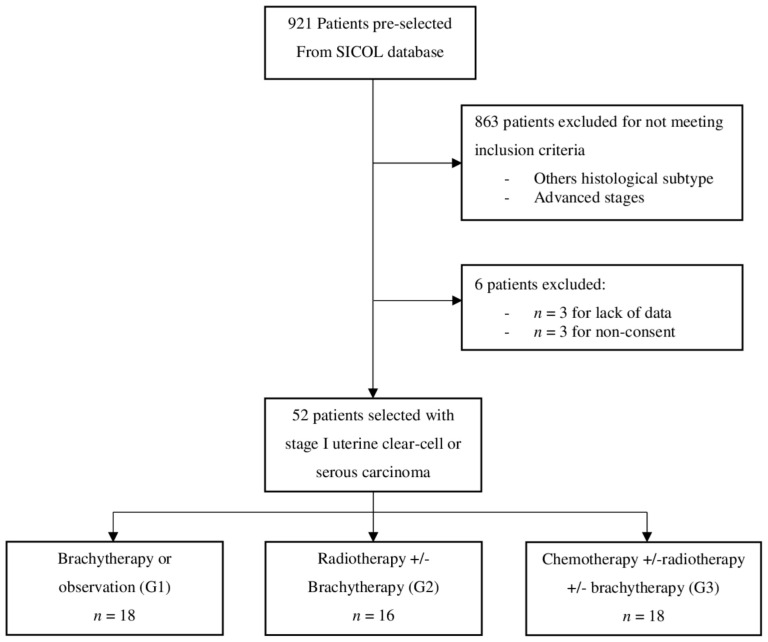
Flowchart.

Fifty-two patients with early stages (IA or IB) UCCC or UPSC were included in the study (Figure 1, Flowchart). The median follow-up was 45.3 months (IC 95%; 34.2–54.8). Patients were stratified into three groups based on the adjuvant treatment: 18 patients were included in G1, 16 in G2, and 18 in G3. 

There was no statistically significant difference between the three groups except for the age at diagnosis and the patients’ performance status (Table 1). Patients were significantly older in G1 with a median age of 76 years and 61.1% (11 patients) over 70 years old compared to median age of 70 years in G2 with 50% over 70 years (8 patients) and 61 years in G3 with only one patient over 70 years. In contrast, concerning the performance status (PS), patients were in a better overall condition in G2 with 81.3% (13 patients) having a PS 0, and in G3 with 86.9% (16 patients) having a PS 0 compared to G1 in which only 50% (9 patients) were PS 0.

### 3.1. Pathological Characteristics

Pathological characteristics are shown in Table 1. In G3, the rate of serous carcinoma was higher (*n* = 15; 83.3%) than the other groups: 55.6% (*n* = 10) in G1 and 56.3% (*n* = 9) in G2 (*p* = 0.14). Similarly, a higher proportion of positive LVSI was observed in the G3 (*n* = 5; 27.8%) compared to G2 (*n* = 1; 6.3%) and G1 (*n* = 2; 11.1%), (*p* = 0.24). However, there were more FIGO IA stages in G1 and G2 (*n* = 15; 83.3% and *n* = 13; 81.3% respectively) compared to G3 (*n* = 13; 72.2%).

### 3.2. Adjuvant Treatments and Toxicities

The morbidity of adjuvant therapy is highlighted in Table 2. 

#### 3.2.1. Brachytherapy

In G1, ten patients (55.6%) did not undergo adjuvant therapy: advanced age and frailty were the reason in five patients, three patients refused the treatment, one patient was previously treated with chemoradiation for cervical carcinoma, and one patient presented an early vaginal recurrence. In this same group, eight patients (44.4%) underwent brachytherapy, out of which one patient showed a grade 1 complication (cystitis). In the G2, 81.3% (13 patients) underwent brachytherapy.

#### 3.2.2. Radiotherapy

Eight patients (50.0%) presented a radiation-related complication, out of which only one patient presented a grade 2 digestive tract toxicity. In G3, 61.1% (11 patients) also had adjuvant radiotherapy, and 88.9% (16 patients) had additional brachytherapy. Three patients (27.3%) had toxicity due to radiotherapy (only grade 1).

#### 3.2.3. Chemotherapy

Concerning the toxicity of chemotherapy, 83.3% (15 patients) presented with treatment-related complications. The grade 2 toxicities: 3 patients presented hematologic toxicity, 14 skin toxicities, 3 neurologic complications, 1 digestive toxicity, and 1 patient presented an altered overall status. We identified two toxicities (15.3%) of grade 3 (one hematologic and one neurologic).

### 3.3. Survival Analysis and Recurrences

#### 3.3.1. Survival Analysis

**Figure 2:**
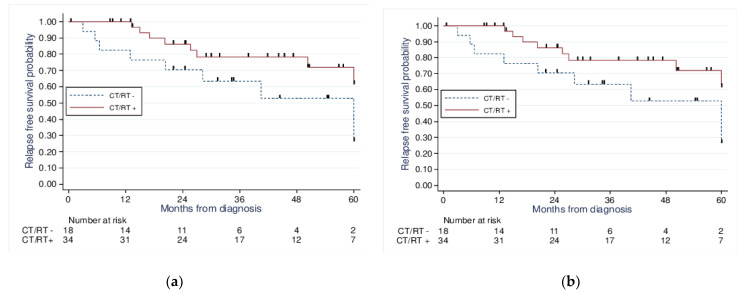
Kaplan−Meier analysis of 5−year overall survival (**a**) (*p* = 0.24) and 5−year progression-free survival (**b**) (*p* = 0.23) for patients with stage I UCCC and USC according to treatment received: CT/RT+ vs. CT/RT−.

Moreover, we performed a survival analysis using the Kaplan–Meier method adjusted to a propensity score according to the adjuvant therapy received. When comparing those treated with chemotherapy and or radiotherapy to those without chemotherapy or radiotherapy (Figure 2), we found no significant improvement in the 5-year OS (71% vs. 52.9%, *p* = 0.24) or 5-year PFS (71.1% vs. 52.9%, *p* = 0.23).

**Figure 3:**
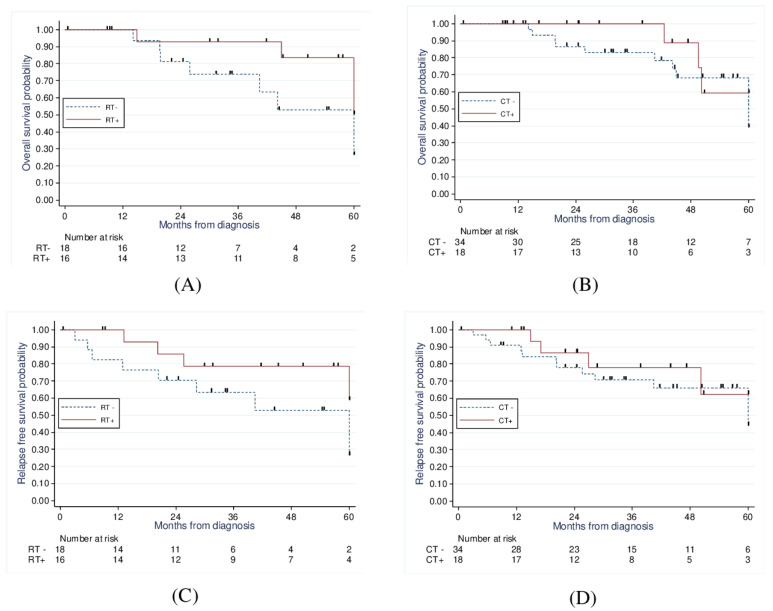
Kaplan−Meier analysis of 5−year overall survival of RT vs. no RT (83.5% vs. 52.8%, *p* = 0.02) (**A**) and CT vs. no CT (59.3% vs. 59.2%, *p* = 0.96) (**B**); and 5−year progression−free survival of RT vs. no RT (78.5% vs. 52.9%, *p* = 0.056) (**C**) and CT vs. no CT (62.4% vs. 66.1%, *p* = 0.84) (**D**).

When comparing those treated with radiotherapy to those without radiotherapy (Figure 3), there was a significant improvement in 5-year OS (83.5% vs. 52.8%, *p* = 0.02) and a strong statistical trend concerning the 5-year PFS (78.5% vs. 52.9%, *p* = 0.056).

Finally, when comparing those treated with chemotherapy to those treated without chemotherapy (Figure 3), we found no significant improvement in 5-year OS (59.3% vs. 59.2%, *p* = 0.97) and 5-year PFS (62.4% vs. 66.1%, *p* = 0.84).

#### 3.3.2. Recurrence Data

Recurrence data are described in Table 1. Patients with brachytherapy only or no adjuvant treatment presented more recurrences (33.3%) compared to the radiotherapy group (18.7%) or the chemotherapy group (16.7%). We found that the recurrences were mainly extra-pelvic with peritoneal carcinomatosis in the three groups. A total of 10 out of the 12 patients who recurred died. We also analyzed OS after recurrence in our overall population. We found a median OS of 16.6 months and a 5-year OS rate of 10.3%.

## 4. Discussion

The most appropriate adjuvant treatment for surgically resected stage I UPSC and UCCC is still controversial due to the lack of reliable scientific data. Most recommendations come from a subgroup analysis of randomized trials such as the PORTEC 3 trial [21].

This study showed that adjuvant RT significantly improved the 5-year OS (*p* = 0.02) with a trend towards improving 5-year PFS (*p* = 0.056) compared to either brachytherapy or observation. Surprisingly, adjuvant CT did not seem to improve either 5-year OS (*p* = 0.97) or PFS (*p* = 0.84). However, it must be considered that, although not significant, patients in the CT group presented known poorer prognostic factors [22]: 83.3% of serous histology, out of which 27.8% presented (≥1) LVSI, and 27.8% were FIGO IB stages. This suggests that CT may have contributed to maintaining comparable survival outcomes in this group.

When analyzing treatment-related toxicities, CT was associated with a higher rate of severe adverse effects (83.3%): All patients presented at least one grade 2 toxicity (14 patients had grade 2 skin toxicity), and two patients (15.3%) presented grade 3 toxicities (1 hematologic and one neurologic). Half of the patients presented toxicities in the RT group, with only one grade 2 toxicity reported. In the brachytherapy group, only one patient presented grade 1 toxicity. The age at diagnosis was the only significant prognosis factor found in univariate analysis.

In a retrospective study, Armbruster et al. showed no statistical difference in the survival outcomes between the observation group and any adjuvant therapy group in patients with FIGO stage I and II mixed or pure UCCC [10]. In 2013, Yechieli et al. studied the effect of adjuvant radiation in patients with early-stage type II (UPSC and UCCC) endometrial carcinoma. They showed that RT significantly improved PFS, and there was a trend toward improved 5-year OS regardless of the use of CT or not [11]. Later in 2020 and 2021, two studies suggested that vaginal brachytherapy associated with adjuvant chemotherapy may be a suitable option for patients with stage I UPSC who underwent surgical staging especially those with poor prognostic factors [12,23]. Other retrospective studies showed no difference in survival outcomes between observation and adjuvant therapy groups. In 2016, Velker et al. found that observation only was an acceptable choice in patients with stage IA UPSC or UCCC [9].

Similarly, in 2003, Huh et al. evaluated the survival outcomes of UPSC stage I patients treated surgically according to the adjuvant therapy received. Their findings showed no statistical difference in 5-year DFS and 5-year OS of patients treated with adjuvant radiation compared to those who underwent close surveillance [24]. However, it must be noticed that only 12% of patients received adjuvant chemotherapy in this study, and there were no recurrences in this cohort of patients, suggesting that CT may have a potential benefit on survival. Due to the small number of patients in this group, survival results may be biased.

UPSC and UCCC are aggressive tumors with poor survival outcomes and a propensity to extrauterine spread compared to the endometrioid subtype [25,26,27]. According to the Hamilton et al. study, UPSC seems to have even poorer survival than the UCCC with a 5-year disease-specific survival of 55% and 68%, respectively, compared to 77% for the G3 endometrioid carcinoma [25]. Our study found that OS after recurrence was 16.6 months and a 5-year rate of 10.3%, which shows the importance of appropriate adjuvant therapy after surgery.

In the latest European guidelines (2021), brachytherapy or observation only can be considered for stage IA without myometrial invasion. RT with concurrent adjuvant CT, or sequential CT, and RT are recommended for high-risk carcinoma, but the benefit of added CT is unclear for UCCC [17]. Several other studies found a benefit of RT and/or CT on survival. In 2019, Randall et al. performed a phase III trial that concluded the non-superiority of brachytherapy followed by three chemotherapy cycles compared to RT in high-intermediate and high-grade early-stage carcinoma (15% of UPSC and 5% of UCCC in each group). Chemotherapy has, however, increased the rate and severity of treatment-related toxicity. In this study, no pelvic or para-aortic recurrences were found among patients with UCCC [28]. In 2018, the PORTEC-3 clinical trial showed that chemoradiation improved the 5-year PFS for high-risk endometrial carcinoma patients, especially those presenting FIGO stages III.

In contrast, 5-year OS was not significantly enhanced with chemoradiation than RT only [21]. In 2009, the Fader et al. retrospective study found that CT significantly improved PFS and decreased the risk of recurrence in early-stage UPSC regardless of the RT [13]. A survival improvement was also seen with CT in patients with stage IB UPSC in the study performed by Cham et al. [14].

Concerning the UCCC and considering its less important chemosensitivity, some studies suggest that exclusive adjuvant RT may be sufficient. The latest 2021 European guidelines highlighted that the benefit of added chemotherapy is still unclear in stage I–II UCCC [17]. In 2017, Nguyen et al. showed an improvement of OS and PFS with RT in early-stage UCCC. However, CT did not have a statistically significant effect on OS and PFS [15]. Thomas et al. showed in their retrospective analysis that UCCC might behave less aggressively than UPSC, at least in the early stages, with a 5-year survival rate of 79% in patients with stage I UCCC [6]. Similar survival rates were reported by Creasman et al. [29]. Thomas et al. found that RT was associated with improved survival outcomes and reduced pelvic sidewall and vaginal recurrences, especially in patients with suboptimal lymphatic assessment or metastatic lymph nodes. RT was also an independent predictor of PFS and OS on multivariate analysis.

Considering both literature data and our results, we suggest that adjuvant therapy is needed in these aggressive tumors. RT may be sufficient in patients with UCCC. However, given the severity of UPSC and its propensity to extrauterine and lymphovascular spread [5], adding an adjuvant CT to RT may be considered in patients with stage I UPSC.

Another result that should be discussed is treatment toxicity. Our study found that chemotherapy was responsible for more severe toxicities (grades 2 and 3) than radiation therapy (external beam or brachytherapy). These results are in accordance with the PORTEC-3 trial by de Boer et al., which showed that chemotherapy significantly increases the risk of severe adverse events and decreases the level of patient’s quality of life, compared to RT alone in women with high-risk endometrial cancer [30]. The same results are found in Randall’s phase III trial in 2019 [28]. This increased CT toxicity must be considered in the adjuvant treatment decision and adapted according to patients’ characteristics and histological results.

The limitations of our study are mainly its retrospective nature spanning an extended period, potentially involving data collection bias. Patients’ data were reviewed over 17 years, and treatment patterns may have changed during this period. Moreover, assessing patients with FIGO stage I tumors only of rare histology in a single center leads to a small sample size and, eventually, a lack of statistical power.

The strength of our study is that we are presenting one of the largest European cohorts in the literature over a long period. Uterine serous and clear cell carcinomas are rare histologies, frequently mixed with other subtypes with better prognoses in literature. Only a few articles have focused on these subtypes.

## 5. Conclusions

In conclusion, our study suggests that radiation therapy alone may be sufficient as adjuvant therapy in patients with stage I UPSC or stage I UCCC, especially in the elderly or frail population that do not present poor histological prognostic factors. Adjuvant chemotherapy can be considered for patients with preserved overall condition, in the UPSC subtype, or if patients present poor prognostic factors (LVSI, FIGO IB). Further studies with prospective design and larger population samples are needed to confirm these results differentiating UCCC from UPSC.

## Figures and Tables

**Table 1:** Patients’ characteristics.

Characteristics	Brachytherapy or Observation (G1)		Radiotherapy +/− Brachytherapy (G2)		Chemotherapy +/− Radiotherapy +/− Brachytherapy (G3)		Global		*p*-Value
	*n* = 18		*n* = 16		*n* = 18		*n* = 52		
Age									0.005
Median—(Range)	76.0	(33.0; 86.0)	70.0	(56.0; 83.0)	61.0	(51.0; 73.0)	65.0	(33.0; 86.0)	
Mean—SD	70.7	13.4	61.3	8.9	66.8	5.4	66.9	10.5	
Age-group									0.001
≤70	7	38.9%	8	50.0%	17	94.4%	32	61.5%	
>70	11	61.1%	8	50.0%	1	5.6%	20	38.5%	
Mean—SD	29.1	6.0	29.2	5.5	30.1	7.3	29.5	6.2	
Missing data	2				1		3		
OMS Status									0.074
0	9	50.0%	13	81.3%	16	88.9%	38	73.1%	
1	7	38.9%	2	12.5%	2	11.1%	11	21.2%	
2	1	5.6%	1	6.3%	0	0.0%	2	3.8%	
3	1	5.6%	0	0.0%	0	0.0%	1	1.9%	
OMS Status-group									0.028
grade 0	9	50.0%	13	81.3%	16	88.9%	38	73.1%	
grade 1, 2, or 3	9	50.0%	3	18.8%	2	11.1%	14	26.9%	
Time between biopsy and surgery (weeks)									0.856
Median—(Range)	7.4	(2; 14.9)	6.7	(2.7; 16.4)	7.4	(3.0; 11.7)	7.4	(2.0; 16.4)	
Mean—SD	7.5	3.1	7.9	3..6	7.4	2.1	7.76	2.9	
Missing	1		1		0		2		
Lymphadenectomy									0.163
No	7	38.9%	3	18.8%	2	11.1%	12	23.1%	
Yes	11	61.1%	13	82.2%	16	89.9%	40	76.9%	
Pelvic	6	33.3%	6	37.5%	4	22.2%	16	30.8%	
Lumbo-aortic pelvic	5	27.8%	7	43.8%	12	66.7%	24	46.2%	
Restaging									0.109
No	16	94.1%	10	66.7%	10	66.7%	36	76.6%	
Yes	2	5.9%	5	33.3%	5	33.3%	11	24.4%	
Lombo-aortic	1	5.9%	1	6.7%	3	20.0%	5	10.6%	
Pelvic and lombo-aortic	0	0.0%	4	26.7%	2	13.3%	6	12.8%	
Missing data	1		1		3		5		
Post-operative complications									0.336
No	17	94.4%	13	81.3%	15	83.3%	45	86.5%	
Yes									
grade 1	1	5.6%	0	0.0%	0	0.0%	1	1.9%	
grade 2	0	0.0%	2	12.5%	1	5.6%	3	5.8%	
grade 3	0	0.0%	1	6.3%	2	11.1%	3	5.8%	
Type of surgical complications									0.829
Lymphoedema	1	100.0%	1	33.3%	0	0.0%	2	28.6%	
Lymphocele	0	0.0%	1	33.3%	0	0.0%	1	14.3%	
Infectious	0	0.0%	0	0.0%	1	33.3%	1	14.3%	
Urologic (fistula, urinoma, urethral wound)	0	0.0%	1	33.3%	2	66.7%	3	42.9%	
Histologies									0.141
Clear-cell	8	44.4%	7	43.8%	3	16.7%	18	34.6%	
Serous	10	55.6%	9	56.3%	15	83.3%	34	65.4%	
Tumor size (mm)									0.469
Median—(Range)	22.0	(6.0; 60.0)	30.0	(6.0; 65.0)	40.0	(9.0; 65.0)	32.0	(6.0; 65.0)	
Mean—SD	30.0	16.1	32.8	16.2	38.1	18.3	33.9	17.0	
Missing	3		3				6		
Tumor size—group									0.417
≤20 mm	7	38.9%	4	25.0%	5	27.8%	16	30.8%	
>20 mm	11	61.1%	12	75.0%	13	72.2%	36	69.2%	
Figo on operating room									0.763
IA	15	83.3%	13	81.3%	13	72.2%	41	78.8%	
IB	3	16.7%	3	18.8%	5	27.8%	11	21.2%	
LVSI									0.243
0–1	16	88.9%	15	93.8%	13	72.2%	44	84.6%	
>1	2	11.1%	1	6.3%	5	27.8%	8	15.4%	
Recurrences									
Vaginal	2	11.1%	1	6.3%	0		3	5.8%	
Extra pelvic	2	11.1%	1	6.3%	1	5.6%	4	7.7%	
Pelvic + peritoneal carcinosis	2	11.1%	1	6.3%	2	11.1%	5	9.6%	

**Table 2:** Treatments and toxicities.

Characteristics	Brachytherapy or Observation (G1)		Radiotherapy +/− Brachytherapy (G2)		Chemotherapy +/− Radiotherapy +/− Brachytherapy (G3)		Global	
	*n* = 18		*n* = 16		*n* = 18		*n* = 52	
No adjuvant treatment	10	55.6%	0	0.0%	0	0.0%	10	19.2%
Adjuvant Brachytherapy	8	44.4%	13	81.3%	16	88.9%	37	71.2%
Dose (Gray)								
6.2	0	0.0%	13	100.0%	9	56.3%	22	59.5%
24.8	8	100.0%	0	0.0%	7	43.8%	15	40.5%
Toxicity associated with Brachytherapy								
No	7	87.5%	13	100.0%	16	100.0%	36	97.3%
Yes	1	12.5%	0	0.0%	0	0.0%	1	2.7%
Adjuvant Radiotherapy	0	0.0%	16	100.0%	11	61.1%	27	51.9%
Dose (Gray)								
45	NA	NA	13	81.3%	7	63.6%	20	74.1%
46	NA	NA	0	0.0%	1	9.1%	1	3.7%
50	NA	NA	0	0.0%	1	9.1%	1	3.7%
50.4	NA	NA	3	18.8%	2	18.2%	5	18.5%
Toxicity associated with RT (all toxicities combined)								
No	NA	NA	8	50.0%	8	72.7%	16	59.3%
Yes	NA	NA	8	50.0%	3	27.3%	11	40.7%
Digestive			6	37.5	3	27.3%	9	33.3%
grade 2	NA	NA	1	6.2%	0	0.0%	1	11.1%
Renal and urinary			4	25.0%	2	18.2%	6	22.2%
grade 2	NA	NA	0	0.0%	1	9.1%	1	16.7%
Gynaecological			1	6.3%	1	9.1%	2	
grade 2	NA	NA	0	0.0%	1	9.1%	1	50.0%
Adjuvant chemotherapy	0	0.0%	0	0.0%	18	100.0%	18	34.6%
Type of Chemotherapy								
Carboplatin + Taxol	NA	NA	NA	NA	16	88.9%	16	88.9%
Cisplatin	NA	NA	NA	NA	2	11.1%	2	11.1%
Toxicity associated with CT								
No	NA	NA	NA	NA	3	16.7%	3	16.7%
Yes	NA	NA	NA	NA	15	83.3.%	15	83.3.%
Hematological					6	33.3%	6	33.3%
grade 2	NA	NA	NA	NA	3	16.7%	3	16.7%
grade 3	NA	NA	NA	NA	1	5.6%	1	5.6%
Cutaneous					14	77.7%	14	77.7%
grade 2	NA	NA	NA	NA	14	77.7%	14	77.7%
Neurological					7	39.9%	7	39.9%
grade 2	NA	NA	NA	NA	3	16.7%	3	16.7%
grade 3	NA	NA	NA	NA	1	5.6%	1	5.6%
Digestive					2	11.1%	2	11.1%
grade 2	NA	NA	NA	NA	1	5.6%	1	5.6%
Altered general condition					7	39.9%	7	39.9%
grade 2	NA	NA	NA	NA	1	5.6%	1	5.6%

## Data Availability

The data presented in this study are available on request from the corresponding author.
